# Plasticity and constraints on fatty acid composition in the phospholipids and triacylglycerols of *Arabidopsis* accessions grown at different temperatures

**DOI:** 10.1186/1471-2229-13-63

**Published:** 2013-04-17

**Authors:** Anushree Sanyal, Craig Randal Linder

**Affiliations:** 1 Section of Integrative Biology, School of Biological Sciences, University of Texas at Austin, Austin, TX, 78712, USA; 2 Department of Agronomy, College of Agricultural and Life Sciences, University of Wisconsin-Madison, Madison, WI, 53706, USA

**Keywords:** Seed oil evolution, Fatty acid, Triacylglycerols, Phospholipids, *Arabidopsis*

## Abstract

**Background:**

Natural selection acts on multiple traits in an organism, and the final outcome of adaptive evolution may be constrained by the interaction of physiological and functional integration of those traits. Fatty acid composition is an important determinant of seed oil quality. In plants the relative proportions of unsaturated fatty acids in phospholipids and seed triacylglycerols often increases adaptively in response to lower growing temperatures to increase fitness. Previous work produced evidence of genetic constraints between phospholipids and triacylglycerols in the widely studied *Arabidopsis* lines Col and L*er*, but because these lines are highly inbred, the correlations might be spurious. In this study, we grew 84 wild *Arabidopsis* accessions at two temperatures to show that genetic correlation between the fatty acids of the two lipid types is not expected and one should not influence the other and seed oil evolution and also tested for the adaptive response of fatty acids to latitude and temperature.

**Results:**

As expected no significant correlations between the two lipids classes at either growing temperature were observed. The saturated fatty acids and erucic acid of triacylglycerols followed a significant latitudinal cline, while the fatty acids in phospholipids did not respond to latitude as expected. The expected plastic response to temperature was observed for all the triacylglycerol fatty acids whereas only oleic acid showed the expected pattern in phospholipids. Considerable phenotypic variation of the fatty acids in both the lipid types was seen.

**Conclusion:**

We report the first evidence supporting adaptive evolution of seed triacylglycerols in *Arabidopsis* on a latitudinal cline as seen in other species and also their plastic adaptive response to growing temperature. We show that as expected there is no genetic correlations between the fatty acids in triacylglycerols and phospholipids, indicating selection can act on seed triacylglycerols without being constrained by the fatty acid requirements of the phospholipids. Phospholipid fatty acids do not respond to latitude and temperature as seen elsewhere and needs further investigation. Thus, the adaptive response of *Arabidopsis* and the genetic tools available for manipulating *Arabidopsis*, makes it an excellent system for studying seed oil evolution and also for breeding seed oil crops especially the *Brassica* species.

## Background

Adaptive evolution is subject to a number of constraining factors that can prevent a species from evolving an optimal phenotype for a particular selective pressure. Adaptation can be constrained by available genetic variation [1,2], and developmental [3], biomechanical-chemical [4-6], and functional [1,7] factors. Such constraints can impose qualitative and quantitative limits on adaptation [1,3,4,8,9]. Further, natural selection acts upon the whole organism rather than on isolated traits, and thus, adaptive evolution may be constrained by the interaction between traits that are functionally or physiologically integrated. In fact, several studies have revealed that natural selection often involves tradeoffs among competing functions and places limits on the course or outcome of adaptive evolution [7,10,11].

Most seed plants at the time of seed production have two major fatty acid (FA) sinks: the triacylglycerols (TAGs) and the phospholipids (PLs). For the most part, TAGs are highly seed specific, occasionally also being produced in large quantities for fruits, e.g., in the mesocarp of avocado and oil palm, whereas PLs are produced in every cell for production and maintenance of membranes [12]. In plants, FAs are synthesized *de novo* in the plastid and modified there and in the endoplasmic reticulum (ER). During synthesis in the plastids, all FAs are initially saturated, with unsaturated FAs being produced from these saturated FAs by desaturases [13]. Generally, plastid-generated FAs are no longer than 18 carbons and have at most a single degree of desaturation. The majority of PL and TAG are assembled in the ER using FAs exported from the plastid [14,15]. In the ER, FAs may be elongated and more highly desaturated before they are incorporated into TAGs and PLs [16-18]. Both PLs and TAGs are synthesized by adding FAs to a glycerol backbone.

Evidence indicates that variation in the FA composition of both TAGs and PLs has played a role in the adaptive evolution of each to temperature [19-21]. The relative proportions of saturated and unsaturated FAs in seed TAGs often shift from high proportions of saturated FAs at low latitudes—where germination temperatures are higher—to lower proportions at higher latitudes—where germination temperatures are lower [19]. In the case of PLs, studies have shown that when temperatures drop, many plants plastically increase the proportion of unsaturated FAs in PLs, which is likely an adaptive plastic response to maintain the flexibility of cellular and other membranes during cold periods [20,21]. Thus, FA composition in seeds and leaves seem to be under strong selection by temperature at both micro- and macro-evolutionary levels and affects the melting point and adaptation to different temperature regimens. Hence, it will be important to identify chromosomal regions responsible for local adaptation and then characterizing the underlying genes as this information will be of immense importance for understanding and enhancing the fitness of the plants in different environmental conditions, breeding oil seed crops, understanding seed oil evolution and for selecting genotypes for producing good quality oil in *Brassica* and other species.

Since the FAs for TAGs and PLs are derived from a common source, selection to optimize each lipid class’s FA composition may be constrained by the selective pressures on the other class. Our understanding of the genetics underlying the regulation of FA production has made considerable progress due to studies by Ohlrogge and colleagues and other groups [22-26]. However, it is not immediately clear how use of particular FAs in PLs and TAGs might affect each lipid class’s FA composition. The outcome of selection could depend heavily on the mechanisms for producing the different pools of FAs. For example, during cooler summers, increased demand for unsaturated FAs in the PLs could limit the availability of unsaturated FAs in seed TAGs, causing the proportion of saturated FAs in TAGs to be higher than is optimal. Alternatively, if increased demand for unsaturated FAs in the PLs unregulated production of unsaturated FAs, the net effect on the seed TAGs might be negligible or could even increase the relative amount of unsaturated fatty acids in them. In some plant species, the FA composition of TAGs is decoupled from changes in the FA composition of PLs [27-29], but this is not universal.

Tradeoffs between the FA compositions of seed TAGs and PLs may also depend on the seasonal germination and maturation time of the seeds. Seed germination in temperate plants often occurs in spring, but those seeds are most often provisioned with TAGs in summer when seeds are maturing [30,31]. The demands for particular FAs in PLs during the period of seed maturation may conflict with the most appropriate FA composition in the TAGs for optimal germination in the spring. In addition, annual variation in summer temperatures might cause the relative amounts of saturated and unsaturated FAs in PLs to vary in different years, further complicating the ability of selection to optimize the FA composition of TAGs. The degree to which PL and TAG production are coupled in *Arabidopsis thaliana* is not known. *A. thaliana* produces FAs for TAGs and PLs that vary widely in their length, degrees of desaturation and melting points (Table 1). Some evidence indicates that the FA composition of its seed TAGs might be constrained by FA use in PLs, but this is based upon only the inbred Columbia (Col) and Landsberg *erecta* (L*er*) lines [32-34]. However, the leaf PL FA composition is not supposed to have any correlation with seed TAG FA composition. Because, the correlation could be an artifact of the low genetic variation in the lines, we used 84 wild *A. thaliana* accessions that were collected over a wide latitudinal gradient in Europe (15-66° N), to study whether the apparent tradeoffs seen in Col and L*er* hold more generally. We also investigated whether a plastic response to temperature in PLs affects the FA composition of seed TAGs by growing all 84 accessions at two temperature regimens. Finally, we tested the strength of the correlations of the individual FAs within PLs and within TAGs, and between PLs and TAGs.

**Table 1:** **Fatty acids in the triacylglycerols and phospholipids of *Arabidopsis***

**Fatty acid**	**Location**	**Symbol**	**Melting point [°C]**
Palmitic	PL, TAG	16:0	62.9
Palmitoleic	PL	16:1	−0.1
Stearic	PL, TAG	18:0	69.5
Oleic	PL, TAG	18:1	13.4
Linoleic	PL, TAG	18:2	−5
Linolenic	PL, TAG	18:3	−10
Arachidic	TAG	20:0	75.3
Eicosenoic	TAG	20:1	25
Eicosadienoic	TAG	20:2	0
Erucic	TAG	22:1	33.5

## Results

The nine individual FAs in seed TAGs and the five individual FAs in PLs showed continuous phenotypic variation. In the TAGs, we observed extensive variation in the proportions of five of the FAs that is 16:0 (5.5-10.2%), 18:1 (5–17.8%), 18:2 (23–37.8%), 18:3 (15-27%) and 20:1(16.4-27.5%) and less than 5% variation in the proportions of 18:0, 20:0, 20:2 and 22:1. We also observed considerable variation in the proportions of 16:0 (10-37%), 16:1(1.5-10%), 18:0 (3-22%), 18:1 (1-9%), 18:2 (7.5-17.5%) and 18:3 (12.6-56%) in the PLs.

### Latitudinal variation in TAGs and PLs

**Figure 1:**
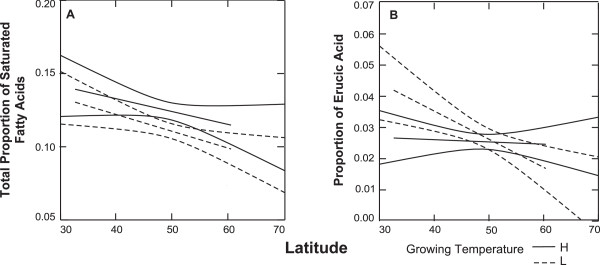
**Regressions of proportions of FAs in TAGs on latitude at high and low temperature treatments.** Only FAs that had a significant cline for at least one growing temperature are presented. High growing temperature regressions are solid lines and low growing temperature regressions are dotted lines. 95% confidence intervals are shown. (**A**) Total saturated FAs (16:0 + 18:0 + 20:0) in TAGs; (**B**) Relative proportion of 22:1in TAGs.

The relative proportion of total saturated FAs in TAGs decreased significantly with increasing latitude when plants were grown at low temperatures (q = 0.007, BH-FDR = 0.024, r^2^ = 0.191, Figure 1A, Table 2). At high temperature, the slope trended in the same direction but was non-significant when controlled for multiple tests, albeit marginally for one test (q = 0.056; BH-FDR = 0.192,Table 2). The slopes of the high and low temperature regressions were nearly identical, with the relative proportions of total saturated FAs in TAGs decreasing by 0.1%/degree of latitude. The relative proportion of total saturated FAs in PLs showed no significant relationship with latitude when corrected for multiple comparisons for both growing temperatures (q ≥0.347, BH-FDR ≥0.347).

**Table 2:** Direction, rate and significance of latitudinal changesin FA proportions in TAGs at high and low growth temperatures

**Trait**	**Direction**^**a**^		**High**^**b**^			**Low**^**c**^			**r**^**2d**^		**Slope**^**e**^	
	**High**^**f**^	**Low**	**P-value**	**Q-value**	**FDR**	**P-value**	**Q-value**	**FDR**	**High**	**Low**	**High**	**Low**
Sat	Decrease	Decrease	**0.040**	0.056	0.192	**0.002**	**0.007**	**0.024**	0.089	0.191	−0.001	−0.001
16:0			0.099	0.059	0.203	0.074	0.059	0.203	0.058	0.068	0.000	0.000
18:0			0.371	0.144	0.495	0.056	0.056	0.192	0.017	0.077	0.000	0.000
18:1	Decrease		**0.048**	0.056	0.192	0.924	0.268	0.924	0.063	0.000	−0.001	0.000
18:2	Increase		**0.033**	0.056	0.192	0.226	0.099	0.341	0.095	0.032	0.001	0.001
18:3			0.110	0.059	0.203	0.428	0.149	0.513	0.054	0.014	0.001	0.001
20:0			0.175	0.087	0.300	0.360	0.144	0.495	0.055	0.042	0.000	0.000
20:1			0.523	0.174	0.598	0.757	0.229	0.790	0.009	0.002	0.000	0.000
20:2			0.227	0.099	0.341	0.107	0.059	0.203	0.032	0.056	0.000	0.000
20:3			0.109	0.059	0.203	0.403	0.148	0.509	0.185	0.089	0.000	0.000
22:1		Decrease	0.675	0.213	0.736	**0.0003**	**0.002**	**0.007**	0.004	0.251	0.000	−0.001
16:0 + 18:0		Decrease	0.097	0.059	0.203	**0.004**	**0.009**	**0.032**	0.059	0.169	−0.001	−0.001

Numbers in bold indicate significance at α = 0.05.

^a^Direction of the change in proportions of FAs as latitude increases.

^b^Significance of the change in proportions of FAs as latitude increases for the high growing temperature treatment.

^c^Significance of the change in proportions of FAs as latitude increasesfor the low growing temperature treatment.

^d^Regressioncoefficients of the change in proportions of FAs.

^e^Rate of change in proportions of FAs per degree of latitude as latitude increases.

^f^Growing temperature.

To assess which of the saturated FAs were most responsible for the latitudinal cline in total saturated FAs in TAGs, we examined the regressions of individual saturated FAs and saturated FAs in combination. None of the individual saturated FAs exhibited a significant latitudinal cline for TAGs (Table 2) even though all of them trended to lower relative proportions at higher latitudes. At low temperature, the sum of palmitic (16:0) and stearic (18:0) FAs—which comprised more than 90% of the total proportion of saturated FAs in TAGs—showed a significant latitudinal cline (q = 0.009, BH-FDR = 0.032, r^2^ = 0.169, Table 1). A similar, but non-significant trend was seen for 16:0 + 18:0 at high temperature.

Finally, we examined the unsaturated FAs in PLs and TAGs to see if any varied significantly with respect to latitude. None of the unsaturated FAs in PLs showed a significant relationship with latitude, whereas in TAGs, only erucic acid (22:1) in plants grown at low temperature varied significantly with latitude (q = 0.002, BH-FDR = 0.007, r^2^ = 0.251, Figure 1B, Table 2), decreasing as latitude increased. On average, the relative proportion of erucic acid decreased by 0.1% /degree of latitude.

### Plastic responses of TAGs and PLs to growing temperature

#### Plasticity in TAGs

The average total saturated FAs in TAGs was higher at higher growing temperatures (high temperature = 0.126, low temperature = 0.112). To further understand this plastic response, we performed four tests to assess the plastic responses of the proportions of total saturated FAs and each of the individual FAs in TAGs of plants grown at high and low temperatures: one-way ANCOVAs that included latitude as a covariate and temperature as a fixed main effect, two-way ANOVA which included temperature and accessions as the factors, one-tailed paired *t*-tests, and chi-square tests.

**Figure 2:**
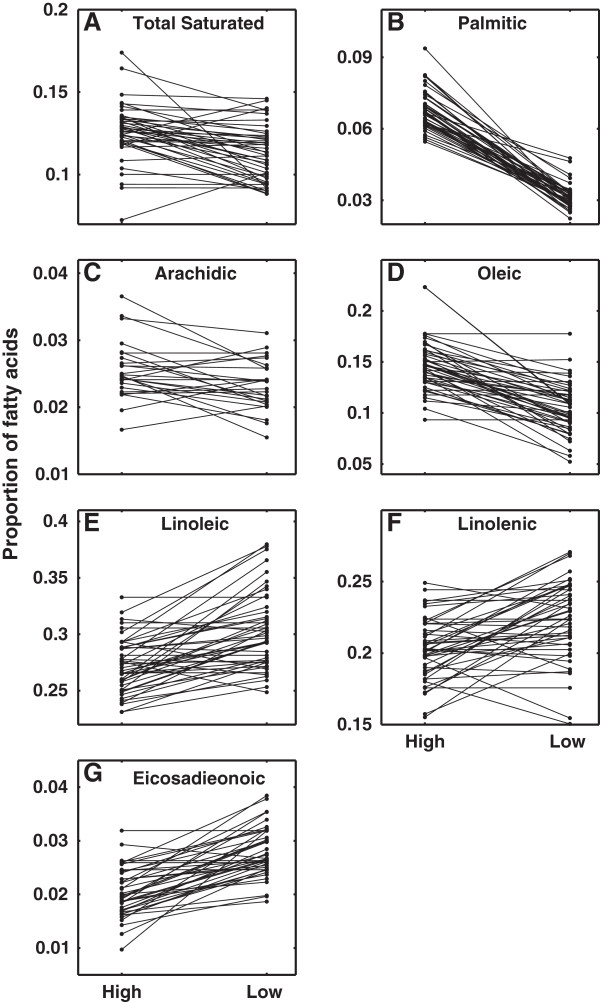
**Reaction norms of the relative proportions of FAs in TAGs.** Only relationships that are significant in all three tests are presented. Each line connects the means of a single lineage grown at high and low temperatures. (**A**) Saturated (**B**) Palmitic (**C**) Arachidic (**D**) Oleic (**E**) Linoleic (**F**) Linolenic (**G**) Eicosadienoic FAs in relation to high and low growth temperature.

For the saturated FAs, the proportion of total saturated and arachidic (20:0) FAs were significantly different at the two growing temperatures for all four tests (total saturated: q < 0.001, BH-FDR < 0.001; arachidic: q ≤ 0.01,BH-FDR ≤ 0.01, Table 3), decreasing at the lower growing temperature. Palmitic acid (16:0) was significant in three of the tests (q < 0.05, BH-FDR < 0.05) with the exception of the two-way ANOVA (not shown in Table 3) where it was nearly significant at FDR 0.09 and trended in the right direction. Although reaction norms did not show a universal response for all of the accessions (Figure 2A-2C), the results from the tests, particularly the chi-square, indicate that many more of them decreased at the lower growing temperature.

**Table 3:** Plastic response of FA proportions in TAGs for plants grown at high and low temperatures

**Trait**	**Proportions **^**a**^		**ANCOVA**		***χ*2**			**Paired-T**			
	**High**	**Low**	**P-value**	**Q-value**	**FDR**	**P-value**	**Q-value**	**FDR**	**P-value**	**Q-value**	**FDR**
Sat^b^	0.126[0.007]	0.112[0.006]	**0.0001**^**c**^	**0.0001**	**0.0002**	**0.0005**	**0.0015**	**0.0015**	**1.07x10**^**-5**^	**2.134x10**^**-5**^	**2.13x10**^**-5**^
16:0	0.072[0.004]	0.068[0.003]	**0.016**	**0.015**	**0.025**	**0.003**	**0.007**	**0.007**	**0.0015**	**0.0026**	**0.0026**
18:0	0.030[0.002]	0.031[0.002]	0.534	0.356	0.484	0.094	0.141	0.141	0.744	0.812	0.812
18:1	0.146[0.008]	0.105[0.003]	**2.3x10**^-**11**^	**1.6x10**^**-10**^	**2.6x10**^**-10**^	**3.84x10**^**-5**^	**0.0002**	**0.0002**	**2.7x10**^**-13**^	**3.20x10**^**-12**^	**3.2x10**^**-12**^
18:2	0.273[0.004]	0.302[0.005]	**4.3x10**^**-6**^	**9.95x10**^**-6**^	**1.64x10**^**-5**^	**0.0005**	**0.0015**	**0.0015**	**1.04x10**^**-7**^	**6.24x10**^**-7**^	**6.24x10**^**-7**^
18:3	0.203[0.005]	0.224[0.003]	**0.0001**	**0.0001**	**0.0002**	**0.005**	**0.01**	**0.01**	**4.45x10**^**-6**^	**1.07x10**^**-5**^	**1.07x10**^**-5**^
20:0	0.023[0.004]	0.012[0.004]	**4.48x10**^**-6**^	**9.95x10**^**-6**^	**1.64x10**^**-5**^	**0.007**	**0.011**	**0.011**	**6.86x10**^**-7**^	**2.74x10**^**-6**^	**2.74x10**^**-6**^
20:1	0.204[0.006]	0.207[0.006]	0.396	0.293	0.484	0.272	0.322	0.322	0.707	0.812	0.812
20:2	0.019[0.001]	0.026[0.001]	**1.25x10**^**-5**^	**2.08x10**^**-5**^	**3.43x10**^**-5**^	**1.38x10**^**-5**^	**0.0002**	**0.0002**	**2.12x10**^**-6**^	**6.35x10**^**-6**^	**6.35x10**^**-6**^
20:3	0.002[0.0003]	0.001[0.0004]	0.998	0.604	0.998	0.661	0.661	0.661	0.520	0.694	0.694
22:1	0.025[0.001]	0.026[0.002]	0.386	0.293	0.484	0.272	0.322	0.322	0.822	0.822	0.822

^a^Means and [standard error] of FA proportions at high and low growing temperatures.

^b^Total proportion of saturated FAs.

^c^All tests that were significant at α ≤ 0.05 are in bold.

For individual unsaturated FAs, all four tests were significant for the relative proportions of oleic (18:1), linoleic (18:2), and eicosadienoic (20:2) FAs at high and low growing temperatures (oleic: q < 0.001, BH-FDR < 0.001; linoleic: q < 0.01, BH-FDR < 0.01; eicosadienoic: q < 0.001, BH-FDR < 0.001, Table 3). Linolenic acid (18:3) was significant for three of the tests (linolenic: q ≤ 0.01, BH-FDR ≤0.01) with the exception of the two-way ANOVA (not shown in Table 3). For each trait, the direction of the reaction norms was not universal for all of the accessions (Figure 2D-2G), but the proportions of linoleic, linolenic and eicosadienoic acids generally increased for the low temperature treatment, and the proportion of oleic acid decreased.

#### Plasticity in PLs

For the FAs in the PLs, the proportions of total saturated, palmitoleic, stearic, oleic and linoleic (Table 4) FAs varied significantly by growing temperature, and only when ANCOVAs and ANOVAs (data not shown in Table 4) were performed (saturated: F_1_, _112_ =9.36, q = 0.005, BH-FDR = 0.05; palmitoleic: F_1_, _112_ = 13.45, q = 0.001, BH-FDR = 0.001; stearic: F_1_, _112_ = 27.6, q < 0.0001, BH-FDR < 0.0001; oleic: F_1_, _112_ = 12.11, q = 0.002, BH-FDR = 0.02; linoleic: F_1_, _112_ = 7.23,q = 0.012, BH-FDR = 0.012), indicating that without correction for latitude, other factors obscured these trends. The trends of the FAs were not in the expected direction because the relative proportions of saturated and stearic acids increased in plants grown at low temperature whereas the relative proportion of palmitoleic and linoleic acids decreased. Only the relative proportion of oleic acid decreased at lower temperatures as expected.

**Table 4:** Plastic response of FA proportions in PLs for plants grown at high and low temperatures

**Trait**	**Proportion**^**a**^		**ANCOVA**			***χ*2**			**Paired-*T *test**		
	**High**	**Low**	**P-value**	**Q-value**	**FDR**	**P-value**	**Q-value**	**FDR**	**P-value**	**Q-value**	**FDR**
Sat^b^	0.294[0.016]	0.337[0.009]	**0.003**^**c**^	**0.005**	**0.005**	0.173	0.403	0.403	0.955	1	1
16:0	0.230[0.016]	0.235[0.010]	0.684	0.798	0.79	0.714	0.833	0.833	0.464	1	1
16:1	0.042[0.007]	0.030[0.002]	**0.0004**	**0.001**	**0.001**	**0.032**	0.110	0.110	0.999	1	1
18:0	0.066[0.003]	0.103[0.005]	**7.7x10**^**-7**^	**5.4x10**^**-6**^	**5.4x10**^**-6**^	**0.032**	0.110	0.110	1	1	1
18:1	0.037[0.006]	0.021[0.003]	**0.0007**	**0.002**	**0.002**	0.267	0.467	0.467	**0.036**	0.288	0.288
18:2	0.139[0.007]	0.120[0.005]	**0.0083**	**0.012**	**0.012**	0.714	0.833	0.833	0.974	1	1
18:3	0.486[0.023]	0.492[0.012]	0.878	0.878	0.878	0.903	0.903	0.903	0.329	1	1

^a^Means and [standard error] of FA proportions at high and low growing temperatures.

^b^Total proportion of saturated FAs.

^c^All tests that were significant at α ≤0.05 are in bold.

#### Correlation of FAs in TAGs and PLs

We investigated the correlations between FAs in TAGs and PLs in plants grown at high and low temperatures to study whether genetic constraints might influence the FA proportions in each sink. We also quantified the correlations between individual FAs within TAGs and PLs to see how the proportions of fatty acids in each affect one another and to provide some insight into the regulation of the relative proportions of fatty acids in each lipid category.

#### Correlations of FAs between TAGs and PLs

**Figure 3:**
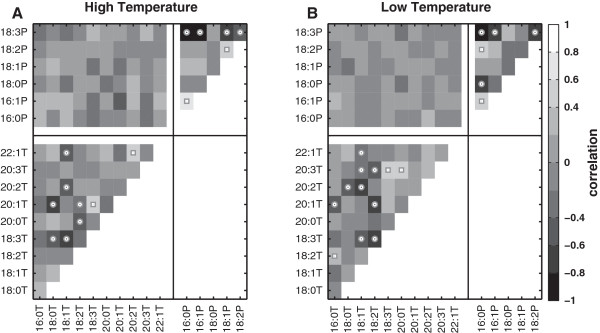
**Correlations among FAs between and within TAGs and PLs.** ‘T’ and ‘P’ indicates FAs present in TAGs and PLs, respectively. Circles and squares in the cells indicate significant negative and positive correlations, respectively, at BH-FDR and q-value =0.05. The numerical bins for the correlations corresponding to the shading are shown in the bar to the right. (**A**) Correlations among FAs in TAGs and PLs for plants grown at high temperature. (**B**) Correlations among FAs in TAGs and PLs for plants grown at low temperature.

As expected we did not find any significant correlations between the FAs of seed TAGs and leaf PLs irrespective of temperature treatment (Figure 3, Tables 5 and 6).

**Table 5:** Correlations of FAs proportions within and between TAGs and PLs in plants grown at high temperature treatment

	**16:0T**^**a**^	**18:0T**	**18:1T**	**18:2T**	**18:3T**	**20:0T**	**20:1T**	**20:2T**	**20:3T**	**22:1T**	**16:0P**^**b**^	**16:1P**	**18:0P**	**18:1P**	**18:2P**
18:0T	0.279														
18:1T	0.151	0.328													
18:2T	−0.150	−0.088	−0.193												
18:3T	−0.320	**−0.423**^**c**^	**−0.636**	−0.330											
20:0T	0.150	0.315	0.069	**−0.411**	−0.096										
20:1T	−0.341	**−0.622**	−0.161	**−0.388**	**0.411**	−0.252									
20:2T	−0.067	0.068	**−0.579**	0.086	0.189	−0.008	−0.192								
20:3T	−0.057	−0.100	0.083	−0.158	−0.067	0.087	0.022	0.025							
22:1T	0.138	0.288	**−0.452**	−0.096	0.025	0.336	−0.237	**0.596**	−0.086						
16:0P	0.259	−0.039	0.240	−0.013	−0.269	−0.104	−0.025	0.025	−0.207	0.023					
16:1P	0.343	0.306	0.213	0.030	−0.301	0.120	−0.441	0.215	−0.275	0.242	**0.788**				
18:0P	−0.015	0.030	−0.239	0.190	0.118	−0.292	−0.056	0.282	−0.030	0.089	−0.160	−0.045			
18:1P	0.132	0.297	0.125	0.143	−0.242	0.154	−0.357	0.024	−0.134	0.138	0.324	0.385	−0.084		
18:2P	−0.106	0.109	0.058	0.017	0.021	0.078	−0.037	−0.178	−0.023	−0.125	0.087	0.089	−0.119	**0.563**	
18:3P	−0.237	−0.128	−0.210	−0.065	0.269	0.045	0.193	−0.056	0.234	−0.067	**−0.887**	**−0.805**	0.011	**−0.638**	−0.449

^a^‘T’ indicates fatty acids in triacylglycerols.

^b^‘P’ indicates fatty acids in phospholipids.

^c^Numbers in bold indicate that the correlation is significant at Q- value and BH-FDR of0.05.

**Table 6:** Correlations of FAs proportions within and between TAGs and PLs in plants grown at low temperature treatment

	**16:0T**^**a**^	**18:0T**	**18:1T**	**18:2T**	**18:3T**	**20:0T**	**20:1T**	**20:2T**	**20:3T**	**22:1T**	**16:0P**^**b**^	**16:1P**	**18:0P**	**18:1P**	**18:2P**
18:0T	−0.145														
18:1T	−0.241	0.060													
18:2T	**0.371**^**c**^	−0.127	0.102												
18:3T	−0.272	−0.034	**−0.501**	**−0.635**											
20:0T	0.098	−0.170	−0.064	−0.342	−0.088										
20:1T	**−0.464**	0.055	0.077	**−0.619**	0.277	−0.040									
20:2T	0.024	**−0.500**	**−0.608**	−0.127	0.339	0.116	−0.043								
20:3T	0.050	−0.088	**−0.399**	**−0.508**	**0.498**	**0.449**	0.063	0.231							
22:1T	0.163	0.202	**−0.366**	−0.064	−0.038	0.132	−0.195	0.210	0.095						
16:0P	0.015	−0.168	0.039	−0.039	−0.096	0.381	−0.159	0.082	0.144	0.058					
16:1P	0.125	−0.168	−0.115	0.240	−0.200	0.163	−0.186	0.143	−0.033	0.033	**0.573**				
18:0P	0.008	0.142	−0.059	−0.008	0.137	−0.242	0.069	−0.025	0.008	−0.066	**−0.650**	−0.259			
18:1P	−0.112	−0.240	0.172	−0.040	−0.032	0.023	0.050	0.017	−0.054	−0.033	0.106	0.090	−0.274		
18:2P	−0.101	0.066	0.024	−0.097	0.080	0.131	−0.005	−0.130	0.057	−0.007	**0.558**	0.262	−0.340	−0.366	
18:3P	0.033	0.130	−0.035	0.050	0.021	−0.303	0.126	−0.028	−0.135	−0.014	**−0.896**	**−0.644**	0.339	−0.025	**−0.687**

^a^‘T’ indicates fatty acids in triacylglycerols.

^b^‘P’ indicates fatty acids in phospholipids.

^c^Numbers in bold indicate that the correlation is significant at Q-value and BH-FDR = 0.05.

#### Correlations among FAs in TAGs

The pairwise correlations among the FAs in TAGs were complex (Figure 3, Tables 5 and 6) and did not have a straightforward interpretation. At high temperature, nine of the correlations were significant when controlled for multiple tests, and at low temperature there were twelve. Of these, only four were significant at both high and low temperature and all of them were negative correlations.

For each class of correlation combinations (saturated:saturated, saturated:unsaturated, unsaturated:unsaturated), the relative numbers of significant positive and negative correlations did not differ from a null expectation of equal numbers whether the data were analyzed by growing temperature or by lumping across growing temperatures (*x*^2^, G-test, and Fisher’s exact test: q ≥ 0.298, BH-FDR ≥ 0.298).

One might expect reactants and products in pathways to be negatively correlated since conversion of one into the other necessarily decreases the reactant and increases the product. When the correlations of single-step reactant-product relationships were examined, no significant correlations were found for the high growing temperature, and one positive and one negative correlation were detected for the low temperature treatment. Allowing one or two steps between reactant and product more clearly fit the hypothesis. At high temperature, four of the significant correlations were negative and none were positive, and at low temperature five were negative and one was positive. If the temperature results are lumped, there are enough values for a chi-square test, which is significant (chi-square = 6.4, df = 1, p = value 0.011). Interestingly, all of the significant cases where 18:1 was the reactant were seen at both temperatures and were all negative.

#### Correlations among FAs within PLs

The pairwise correlations among the FAs in PLs were also complex (Figure 3, Tables 5 and 6). At high temperature, five of the correlations were significant when controlled for multiple tests, and at low temperature there were six. Of these, three were significant at both high and low temperature. One was positive and two were negative.

For each class of correlation combinations (saturated:saturated, saturated:unsaturated, unsaturated: unsaturated), the relative numbers of significant positive and negative correlations did not differ from a null expectation of equal numbers whether the data were analyzed by growing temperature or by lumping across growing temperatures (*x*^2^, G-test, Fisher’s exact test: q ≥ 0.842, BH-FDR ≥ 0.897).

As with the FAs in TAGs, we examined whether there was a consistent pattern of significant negative correlations between PL fatty acid reactants and products one or two steps apart. In all cases, only a small number of reactant-product relationships were significantly correlated, preventing any statistical analysis. Also, in all cases the number of significant positive and negative correlations differed by just one.

## Discussion

### Evidence for a latitudinal cline

As predicted by the adaptive theory [19], *A. thaliana* accessions follow a latitudinal cline for total saturated FA composition in their TAGs (Figure 1) independent of the growing temperature. The relative proportion of total saturated FAs in TAGs of *A. thaliana* accessions decreased with latitude as predicted and in accordance with other species with broad latitudinal distributions [19]. The pattern was clearly significant at low growing temperatures and a nearly significant cline was observed at high growing temperatures.

Compared to prior work with *Helianthus*[19], the scatter around the regression was greater. At least two explanations might account for the difference. First, because the *Helianthus* species studied are self-incompatible [35-38] and *A. thaliana* is not [39-41]. It could be that the higher level of inbreeding in *A. thaliana* reduced genetic variation available to selection for optimizing the relative proportions of saturated and unsaturated FA in TAGs. Second, the much smaller seed size of *A. thaliana* may have promoted longer distance migration of its seeds in transported soil or other material, and the higher rate of gene flow could be countering selection. Since these explanations are not mutually exclusive, both could be operating. A third explanation, that genetic variation was depleted in populations as *A. thaliana*’s range expanded northward after the last ice age (due to repeated founding events) seems less likely because *Helianthus* likely underwent a similar range expansion in North America after the last ice age.

When the FAs were examined individually, only one (erucic acid: 22:1) in TAGs showed significant clinal variation (Table 2). However, all individual TAG FAs either trended in the expected directions based upon their melting points or showed no trend. FAs having melting points above 20°C and showing a trend all decreased at higher latitudes, and all FAs having melting points below 20°C and showing a trend increased at higher latitudes. Special comment needs to be made about erucic acid’s trend to lower levels at higher latitudes. In addition to having a relatively high melting point (33.5°C), erucic acid is also known to be unpalatable to many species of animals [29,42]. Since herbivory generally decreases at higher latitudes [43,44] selection might be stronger for higher levels of erucic acid at lower latitude. Thus, both germination temperature and herbivory could be selecting for the relative proportions of erucic acid.

For the PLs, no clear relationship was observed between latitude and FA composition. This might be expected because of previous work demonstrating adaptive plastic responses to temperature in the FA composition of PLs [20,21]. Adaptive plastic responses would weaken selection on genetically deterministic FA composition. However, we found no evidence for consistent adaptive plastic responses to growing temperature in *A. thaliana* [see below]. Perhaps selection on seed TAGs has been stronger than that on the PLs, but at this time we do not have an explanation for the behavior of the FAs in the PLs.

### Plastic responses of FA composition to growing temperature

In TAGs, palmitic (16:0), arachidic (20:0), oleic (18:1), linoleic (18:2), linolenic (18:3) and eicosadienoic (20:2) FAs responded plastically to growing temperature, with the possible exception of oleic acid all of these FAs responded to growing temperature in directions that would be adaptively plastic. Higher melting point FAs decreased at the lower growing temperature and lower melting point FAs increased. Oleic acid has a relatively low melting point (13.4°C), but it decreased at the lower growing temperature. This may be a consequence of the biosynthetic pathway that links oleic acid with linoleic and linolenic acid. The latter two unsaturated FAs have much lower melting points (see Table 1). Linoleic acid is produced by desaturating oleic acid, and linolenic acid is produced by desaturating linoleic acid. Therefore, plastically lowering the melting point of TAGs by increasing the relative proportions of linoleic and linolenic acid may decrease the relative amount of oleic acid. This conjecture is supported also by the negative correlation between oleic and linolenic acid and the negative correlation between linoleic and linolenic acids (Figure 3).

In contrast, only one of the FAs (oleic acid) of PLs responded plastically to growing temperature as expected based on prior work on adaptive cold tolerance in membrane lipids [20,21] (Table 4). This result is very surprising and deserves further study. Several other studies have demonstrated cold acclimation of plasma membranes in *A. thaliana* when temperatures drop below freezing [45-49], but little has been done at the temperature used for our high temperature treatment. Since *A. thaliana* is a cool weather species, it might grow so consistently under cooler temperatures that selection has been relaxed on plastic acclimation of the FA composition of its PLs, at the temperatures we provided. However, this seems unlikely given the temperatures in Europe when *A. thaliana* is setting seed and the appropriate plastic responses of the TAGs.

One might also conjecture that the adaptive plastic response of the FAs in TAGs is constraining the adaptive plasticity of the PLs. However, the FA composition of the leaf PL and seed TAG is not supposed to have any correlation and the complete lack of significant correlations between any TAG and PL fatty acids (Figure 3) indicates clearly that it is unlikely.

### Correlations of FA composition between and within TAGs and PLs

Prior studies on the L*er* and Col accessions in *A. thaliana* indicated some genetic correlation between the FA compositions of seed TAGs and leaf PLs [32-34]. Our study of a broad latitudinal range of eighty-four wild accessions showed no evidence of these correlations. Thus, revealing that there should not be any correlation observed between the FAs of seed TAGs and leaf PLs. This suggests that evolutionarily each lipid category is largely unconstrained by the other and can evolve independently of other within the constraints of available genetic variation.

The regulation of the relative amounts of FA types in PLs and TAGs is very complex and the subject of much ongoing research [50]. Our analysis of the genetic correlations among pairs of FAs yielded only a little insight into the mechanisms that cause the amounts of some FAs to influence the amounts of other FAs. Within the TAGs we did find some evidence that reactant-product relationships were causing negative relationships between some FA reactants and products sources and sinks of FAs, this potential explanation applied to less than half of the significant correlations among the TAG fatty acids (nine of twenty-one). In the case of the PLs some significant reactant-product correlations were also seen, but on balance, approximately half were positive and half were negative.

### Implications for evolutionary studies

The natural variation observed in this study in 84 accessions of *A. thaliana* and in 360 accessions in a previous study by O’Neill et al. in 2003 [51] revealed extensive variation in TAGs in *A. thaliana.* In addition, since no correlation was observed between the FAs in TAGs and PLs, a significant latitudinal cline was observed for saturated FAs and erucuc acid, and also a plastic response of FAs to temperature was observed, *A. thaliana* will be an ideal system to study seed oil evolution. Preliminary screening of fifteen FA synthesis genes of three of the accessions (L*er*-0, Sha and Col-4) showed that nine of these FA synthesis genes had polymorphisms (FATB, FAD2, FAD3, FAE1.1, FAE1.3, FAE1.4, SAD1, SAD2 and SAD4, [26]). Ten or more polymorphisms were observed in six (FAD2, FAD3, FAE1.1, FAE1.3, SAD1 and SAD2) of these nine genes. The number of polymorphisms observed was greater in the introns, intergenic region within 1000 bp upstream of the promoter and synonymous substitutions in the exon region indicating that selection could be acting on these genes and would be a suitable system to investigate seed oil evolution. Since, the FAs in PLs did not follow the expected patterns; it would also be very useful to find out why the FAs in PLs are behaving differently.

### Implications of breeding seed oil crops

The response of FAs to latitude and temperature and the presence of extensive natural variation suggest that recombinant inbred lines (RILs) can be generated from parents which differ in FA composition to conduct marker-assisted selection and to identify desirable alleles and introduce them into agricultural genotypes for seed oil breeding programs especially the *Brassica* oilseed crops. Preliminary investigation has identified several polymorphisms in FA genes, and modern sequencing technology can help us identify several polymorphisms which will enable us to detect relationships between phenotypic variation and gene polymorphisms in the current germplasm without generating a mapping population. The extensive information of the *Arabidopsis* genome and the availability of several RIL populations has been successfully used to elucidate the flowering time genes, FA desaturases and the glucosinate pathway [26,52-54] and would be very useful to understand the seed oil pathway.

## Conclusions

We report the first evidence supporting adaptive evolution of seed TAGs in *A. thaliana* on a latitudinal cline and the first evidence that the plastic responses of seed TAGs to growing temperature appear to be adaptive. We showed that as expected there were no significant genetic correlations between the FAs in seed TAGs and leaf PLs, indicating that selection can act on seed TAGs without being constrained by the FA requirements of PLs. Because of the many genetic tools available for manipulating *A. thaliana*, it is an excellent system for studying the mechanisms of the evolution of seed oil composition and also for breeding seed oil crops especially the *Brassica* species.

Surprisingly, we found no evidence for adaptive plastic responses of PLs to growing temperature at the temperatures used in our experiments. Given the evidence for adaptive plasticity at cool temperatures, this finding deserves further study.

## Methods

### Plant material

Eighty-four accessions of wild *Arabidopsis thaliana* (Table 7), collected over a wide latitudinal gradient in Europe (15-66°N), were obtained from the Arabidopsis Biological Resource Center [http://www.arabidopsis.org/abrc/]. Since *A. thaliana* occurs within a narrow range, there is more representation between 40-60°N. The number of accessions below 40°N and above 60°N latitude is evenly distributed.

**Table 7:** Geographical location, latitude, longitude and altitude for the 84 accessions

**Accession**	**Latitude**	**Longitude**	**Location**	**Altitude [m]**	**Accession**	**Latitude**	**Longitude**	**Location**	**Altitude [m]**
901	N45	E1-E2	Argentat, France	100-200	6678	N50-N51	E8-E9	Tenne, Germany	400-500
902	N15-N17	W23-W25	CapeVerdiIslands	1200	6680	N47-N48	E5	Dijon, France	300-400
1352	N55-56	E13-E14	Lund, Sweden	1-100	6681	N46-N47	E5	Dijon, France	300-400
1364	N51-N52	E9-E10	Hessen, Germany	200-300	6683	N50-N51	E8	Donsbach, Germany	200-300
1540	N53-N54	W3	Southport, UK	1-100	6684	N51	E13-E14	Dresden, Germany	100-200
6600	N51	E10	Rhon, Germany	200-300	6688	N56	E3	Edinburgh, GB	100-200
6602	N48	E8	Freiburg, Germany	200-300	6693	N51-N52	E12-E13	Eilenburg, Germany	100-200
6604	N51-N52	E4-E5	Antwerpen, Belgium	1-100	6694	N51-N52	E9-E10	Eilershausen, Germany	100-200
6609	N53-N54	E10-E11	Buchen, Germany	1-100	6699	N60	E25	Espoo, Finland	1-100
6616	N41-N42	E3	Blanes, Spain	1-100	6700	N58-N59	E23-E28	Estland, Russia	100-200
6617	N41-N42	E3	Blanes, Spain	1-100	6703	N48	E8-E9	Freiburg, Germany	200-300
6619	N41-N42	E3	Blanes/Gerona, Spain	1-100	6704	N50-N51	E8-E9	Frickhoefen, Germany	300-400
6622	N41-N42	E3	Blanes, Spain	1-100	6714	N50-N51	E8	Gabelstein, Germany	100-200
6626	N49	E16-E17	Brunn, Czech	200-300	6716	N53-N54	E10-E11	Gudow, Germany	1-100
6627	N47-N48	E7-E8	Basel, Switzerland	300-400	6720	N50-N51	E8-E9	Gieben, Germany	100-200
6629	N47-N48	E7-E8	Basel, Switzerland	300-400	6732	N49	E2	La Miniere, France	100
6630	N50	E8-E9	Buchlag, Germany	1-100	6733	N52-N53	E9-E10	Hannover, Germany	1-100
6632	N50-N51	E9-E10	Burghaun, Germany	200-300	6739	N51-N52	E8-E9	Hennetalsperre, Germany	400-500
6660	N28	W15-W16	Canary Islands	1260	6751	N34-N36	E74-E80	Kashmir, India	1580
6665	N54	E35	Chisdra, Russia	100-200	6752	N46-N47	E14-E15	Karnten, Austria	900-1000
6669	N40-N41	W8-W9	Coimbra, Portugal	100-200	6753	N50-N51	E8-E9	Kronberg, Germany	100-200
6674	N37-N38	E15	Catania,Italy	1-100	6754	N55-56	W5-W6	Killean, UK	400-500
6675	N15-N17	W23-W25	CapeVerdiIslands	1200	6761	N51	E7	Koeln, Germany	1-100
6676	N50	E8 - E9	Darmstadt, Germany	100 -200	6764	N51-N52	E6-E7	Krefeld, Germany	1-100
6768	N55-56	W3-W4	Lanark, GB	100-200	6824	N60	E6	Oystese, Norway	1-100
6769	N57-N58	W4-W5	Loch Ness, GB	1-100	6825	N38	E13-E14	Palermo, Italy	1-100
6770	N52-N53	E4-E5	Leiden, Netherlands	1-100	6827	N38	E13-E14	Palermo, Italy	1-100
6775	N50-N51	E8	Limburg, Germany	100-200	6832	N47	E11	Pitztal/Tirol	1000-1500
6780	N50	E19-E20	Lipowiec, Poland	500	6834	N41-N42	E2-E3	Playa de Aro, Spain	1-100
6781	N42	E3	Llagostera, Spain	1-100	6839	N50-N51	E7	Poppelsdorf, Germany	1-100
6784	N48	E0-E1	Le Mans, France	1-100	6841	N50-N51	E8-E9	Frankfurt, Germany	100-200
6788	N46-N47	E3-E4	Lezoux, France	400-500	6848	N56-N57	E34	Rschew, Russia	100-200
6789	N50-N51	E8-E9	Marburg, Germany	200-300	6855	N41-N42	E3	San Feliu, Spain	1-100
6792	N53-N54	E20-E21	Muhlen, Poland	100-200	6864	N53	E12	Stendal, Germany	100-200
6796	N49	E9-E10	Markt, Germany	200-300	6865	N52-N53	E36-E37	Stobowa, Russia	100-200
6799	N33	E23	Martuba, Libya	100-200	6869	N41-N42	E3	Tossa del Mar, Spain	1-100
6800	N50-N51	E8-E9	Merzhausen, Germany	400-500	6876	N45	E7-E8	Turin, Italy	200-300
6803	N50-N51	E9-10	Niederzenz, Germany	200-300	6879	N48	E7-E8	Umkirch, Germany	200-300
6807	N52-N53	E4	Noordwijk, Netherlands	1-100	6892	N52-N53	E9-E10	Wietze, Germany	1-100
6811	N50-N51	E8-E9	Neuweilnau, Germany	100-200	6918	N60-N66	E21-E30	Tenela, Finland	1-100
6816	N50-N51	E8-E9	Oberusel, Germany	100-200	6920	N48-N49	E8-E9	Wildbad, Germany	500-1000
6823	N53-N54	E8-E9	Ovelgoenne, Germany	1-100	6929	N60	E6	Oystese, Norway	1-100

### Growing conditions

Seeds were imbibed by sprinkling them on moist Metromix 200 soil in individual 2.5" pots with 1–3 seeds per pot. Each line was in its own pot. Imbibed seeds were cold treated at 4°C for 4 days to break dormancy and promote uniform germination. All 84 lines were grown under constant light at two different temperature regimens (10°C and 22-25°C) in growth chambers where the temperature was recorded daily. Two replicates were grown per accession in individual pots in a complete randomized block design. The frequency of testing the replicates of all accessions from all latitudes in the two growth chambers were the same to avoid confounding effects of growth chamber and latitude. The accessions were all spring germinating and were not vernalized. Pots were arranged in a 3 X 7 configuration in trays, which were covered with clear plastic domes for moisture retention for the first 7 days. The plants were bottom watered to 0.5", and the water level was monitored daily. Excess water was drained to prevent fungal growth. Plants were fertilized once a week with Peters 20-10-20 fertilizer and trays were rotated every 2 days to minimize position effects. When the plants began to flower, the number of pots per tray was reduced from 21 to 13, and inflorescences were enclosed in plastic sleeves (Aratubes, AS-08, Lehle Seeds) to prevent cross pollination. The plants were allowed to self-pollinate, and seeds were harvested after the siliques ripened and plants had senesced.

### Determination of fatty acid composition of phospholipids

When plants reached the twelve to fourteen leaf stage generally 5–6 days before bolting, four or five fresh, healthy leaves were collected from each accession at both temperature treatments. Phospholipids were extracted using the modified Bligh and Dyer method [55] to produce fatty acid methyl esters (FAMEs). The lipid mixture was dissolved in diethyl ether and then acetone was added to precipitate phospholipids [56]. The FAMEs were resolved on a Hewlett-Packard 5890A gas chromatograph using a 30 m 70% Cyanopropylpolysilphenylene-siloxane capillary column (BP×70, SGE, Inc.) and detected by flame ionization. For all runs, an initial oven temperature of 190°C was maintained for 5.5 min. and then ramped to 240°C at a rate of 7.5°C/min. Ramping was followed by a final time of 0.50 min at 240°C. Injection volume was 2 μl with a 1:100 split ratio, and each sample was injected twice to assess run-to-run variation. The results for each run were compiled and analyzed by HP Chemstation software (version A.04.02) with the proportion of each fatty acid estimated by the area under the curve for each peak as a proportion of the total area under the peaks. Six fatty acids (16:0, 16:1, 18:0, 18:1, 18:2, and 18:3, Table 1) were measured. The identity of the peaks was determined by size standards RM-6 and NHI-F (Supelco), which were run at the beginning of each set of extractions.

### Determination of seed oil composition

The fatty acids present in seed triacylglycerols represents 93% of total fatty acids at 19 days after flowering. So, the fatty acids extracted from seed oils were primarily from triacylglycerols [57]. Seed oil was extracted from 15–20 seeds of each lineage-temperature combination and converted to FAMEs by the Metcalfe and Wang method [58]. The relative proportions of fatty acids were analyzed by using the same methods as the PLs. Ten fatty acids found in *A. thaliana* seed TAGs (16:0, 18:0, 18:1, 18:2, 18:3, 20:0, 20:1, 20:2, 20:3 and 22:1, Table 1) were measured. The peaks were identified using three size standards: RM-6, NHI-F and 189–19 (Supelco). Spot checks on the repeatability of the results within a sample revealed that the average within-sample error was less than 2% of the value of any FA.

### Statistical analyses

#### Testing for latitudinal clines

The null hypothesis we were testing was that there will be no change in the FA composition in TAGs and PLs in *A. thaliana* with latitude. Because previous work on seed TAGs in other species predicted decreasing levels of saturated FAs in the TAGs at higher latitudes, we tested the relationship between latitude and FA composition of TAGs and PLs by performing linear regressions. In addition to performing the regression analyses on the total proportion of saturated FAs in the seed TAGs and PLs, regression analyses were performed on the individual FAs to see whether particular FAs had a latitudinal cline. For both TAGs and PLs, separate analyses were performed on the FAs extracted from the plants grown at each temperature. All regressions were performed using SYSTAT 10.0 [59]. The p-values were adjusted for multiple-hypothesis testing in R (version 2.9.0 alpha) using the q-value false discovery rate (FDR) plug-in [60-62] in the built in function, p-adjust. Default parameters in the q-value module were used except the bootstrap method was used wherever possible. In our analysis we calculated p-value, q-value and FDR, but we considered FDR ≤ 0.05 as significant in our interpretation of the results. Since, there were more number of accessions from certain latitudes; tests were performed with two replicates of equal number of random accessions representing all latitudes to check if the data was unbalanced. No significant difference was found between the two methods in the analyses.

#### Tests for plastic responses to growing temperature on FA composition

The null hypothesis we were testing is that there will be no effect of the growing temperature on the FA composition of TAGs and PLs. To determine whether the growing temperature of the plants influenced the FA composition of TAGs and PLs, three sets of tests were performed. First, differences in the relative proportions of FAs at the two temperature treatments were tested by performing one-way ANCOVAs that included latitude as a covariate, temperature as a fixed main effect, and the relative proportions of each FA and the composite measure of total saturated FAs as the dependent variable. For FAs found in both TAGs and PLs, we also tested for differences in the relative proportions of the FAs in the TAG and PL sinks at the two temperature treatments by performing two-way ANCOVAs that included latitude as a covariate, growing temperature and lipid type as fixed main effects, and the relative proportions of individual/saturated FAs as the dependent variables. We also performed a two-way ANOVA with growing temperature and accessions as the factors and the relative proportions of individual FA and total saturated FAs as the dependent variable. Second, we performed one-tailed, paired *t* tests on the response of the relative proportions of FAs to high and low growing temperatures in PLs and TAGs to compare the differences in the means of the relative proportions of the FAs in PLs and TAGs at different temperatures. Finally, chi-square tests were performed to determine the association between relative proportions of FAs in TAGs and PLs and growing temperature. P-values were adjusted for multiple tests as described in the previous section.

#### Genetic correlations among FAs

We were testing the null hypothesis that there will be no genetic correlation between FAs in TAGs and PLs and also within TAGs and PLs. We analyzed correlations of FA proportions both within PLs and TAGs and between PLs and TAGs in plants subjected to high and low temperature treatments to assess whether genetic correlations among their FAs constrain the ability of the relative levels of the FAs to vary in those sinks. For all the correlation analyses, Pearson’s correlation coefficient (r) was calculated for all pairs of FAs to evaluate the linear relationship between FAs within and between PLs and TAGs. P-values for the correlations were corrected for multiple tests as previously described within TAGs and between TAGs and PLs. We assessed whether particular types of relationships occurred more often than would be expected by chance. The number of significant positive and negative correlations were tallied for the three combinations of FAs: saturated: saturated, saturated: unsaturated and unsaturated: unsaturated, and values were assessed for both temperature treatments separately and with the data lumped across temperature treatments. These observed values were compared with the numbers that would have been expected if it were equally probable to find negative and positive correlations in each category. Expected values among the categories were scaled according to their relative possible occurrences. We calculated Pearson’s chi-square test, the likelihood ratio chi-square test and Fisher’s exact test in Systat 10.0. P-values were adjusted using methods described earlier.

## Abbreviations

(TAG): Triacylglycerol; (PL): Phospholipid; (FA): Fatty acid; (RIL): Recombinant inbred line.

## Competing interests

The authors declare that they have no competing interests.

## Author’s contributions

AS conducted the experiments, the lipid extractions, analyzed the gas chromatograph data and drafted the manuscript. CRL conceived the study, participated in its design, and drafted the manuscript. Both authors read and approved the final manuscript.
